# Elastoviscous Transitions of Articular Cartilage Reveal a Mechanism of Synergy between Lubricin and Hyaluronic Acid

**DOI:** 10.1371/journal.pone.0143415

**Published:** 2015-11-24

**Authors:** Edward D. Bonnevie, Devis Galesso, Cynthia Secchieri, Itai Cohen, Lawrence J. Bonassar

**Affiliations:** 1 Sibley School of Mechanical and Aerospace Engineering, Cornell University, Ithaca, NY, United States of America; 2 Department of Research and Development, Fidia Farmaceutici SpA, Padua, Italy; 3 Department of Physics, Cornell University, Ithaca, NY, United States of America; 4 Department of Biomedical Engineering, Cornell University, Ithaca, NY, United States of America; University of Rochester, UNITED STATES

## Abstract

When lubricated by synovial fluid, articular cartilage provides some of the lowest friction coefficients found in nature. While it is known that macromolecular constituents of synovial fluid provide it with its lubricating ability, it is not fully understood how two of the main molecules, lubricin and hyaluronic acid, lubricate and interact with one another. Here, we develop a novel framework for cartilage lubrication based on the elastoviscous transition to show that lubricin and hyaluronic acid lubricate by distinct mechanisms. Such analysis revealed nonspecific interactions between these molecules in which lubricin acts to concentrate hyaluronic acid near the tissue surface and promotes a transition to a low friction regime consistent with the theory of viscous boundary lubrication. Understanding the mechanics of synovial fluid not only provides insight into the progression of diseases such as arthritis, but also may be applicable to the development of new biomimetic lubricants.

## Introduction

The healthy function of the body’s articular joints has long been considered a remarkable tribological phenomenon. The main bearing surface, articular cartilage, provides low friction (μ < 0.01) and low wear over decades of constant use[1,2]. This superior function of cartilage has been tied to several mechanical factors including, most notably, lubrication by molecules in synovial fluid and interstitial fluid pressurization within the cartilage matrix[2–5]. In a healthy joint, pressurization of the interstitial fluid supports a substantial portion of the normal load, reducing the stresses experienced by solid-solid contact of apposing asperities. This load sharing between fluid and solid states is the main contributor in the very low friction coefficients previously measured[1,6,7]. However, macromolecules in synovial fluid reduce the shear stresses at the asperity contacts[3–5], and associated tissue damage[8] and cell death[9].

The main macromolecules attributed to synovial fluid lubrication are lubricin (also known as Proteoglycan-4 and superficial zone protein) and hyaluronic acid (HA)[4,10]. Lubricin is considered to be the principal boundary lubricant in synovial fluid[5,11]. Found in relatively low concentrations (~200 μg/mL), the molecule’s carboxy-terminus, a hemopexin-like domain, anchors the molecule to the articular surface, and a hydrophilic oligosaccharide brush domain attracts water to the surface, lowering the boundary friction coefficient[12,13]. Further, intra-articular injection of lubricin in animal models of osteoarthritis inhibited degeneration of articular cartilage[14–16], but this therapy has not yet been tested in humans.

Previous studies using HA have been less clear regarding its lubricating ability. There is evidence that this larger molecule, often over 1 MDa (compared to ~220 kDa for lubricin) can reduce the friction coefficient of cartilage. However, the mechanisms by which this molecule lubricates are not fully understood[5,11,17–19]. In fact, some studies suggest that HA has little lubrication benefit at all[3,11]. Despite this confusion, the use of HA in intra-articular injections, commonly referred to as viscosupplementation, is widespread[20,21]. The lack of consensus regarding the lubricating mechanisms of HA can be attributed to the lack of a suitable analysis framework to account to for its viscosity, which is on the order of 100 times more viscous than water for concentrations found in synovial fluid[10,11]. Although synovial fluid can retain its boundary lubricating ability in the absence of HA[22], recently it has been hypothesized that lubricin and HA may work synergistically to promote more effective lubrication[18,19,23,24]. It has however, been challenging to determine the mechanism for this synergistic interaction.

**Fig 1:**
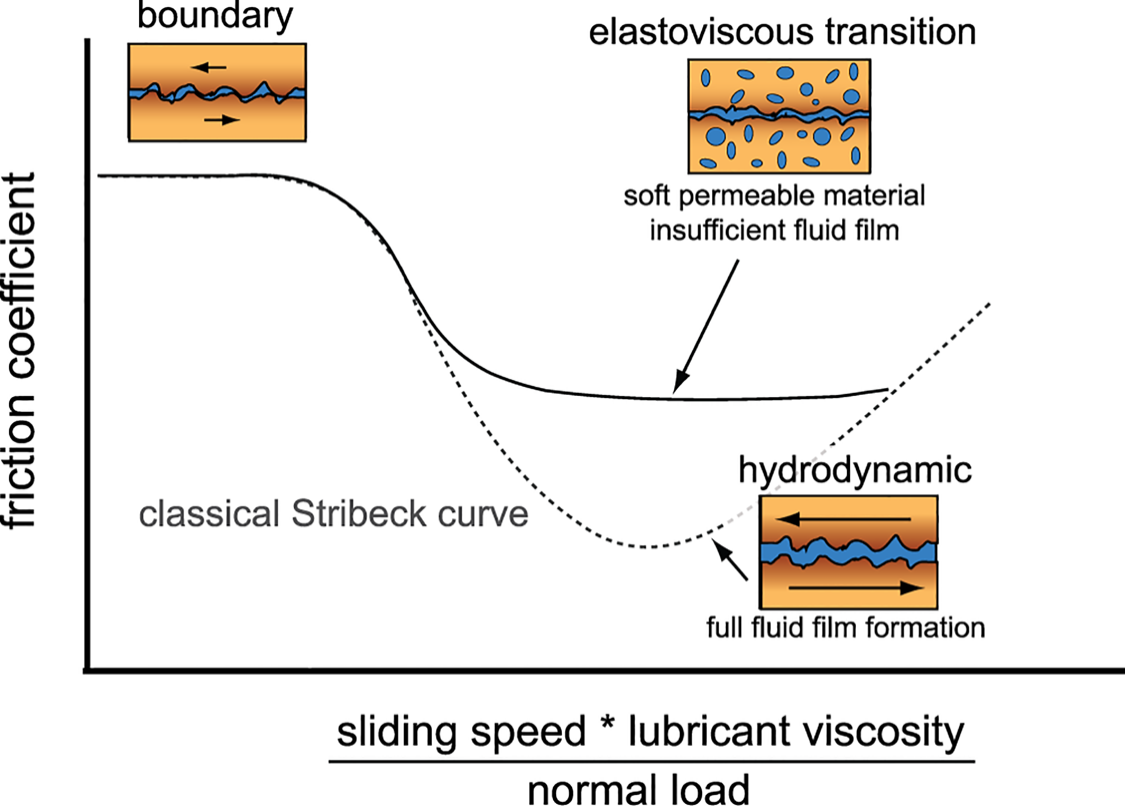
A classical Stribeck curve (dotted line) maps the transition of friction from high (boundary) friction to low (hydrodynamic) friction. For soft, permeable contacts like articular cartilage, a divergence from the classical curve is described by an elastoviscous transition [26] where contact compliance and permeability hinder the transition to hydrodynamic lubrication.

To unambiguously decouple the contributions from viscous lubricants (i.e., HA) and boundary lubricants (i.e., lubricin), it is necessary to map out lubrication modes. Classically, the Stribeck curve revealed distinct modes of lubrication for hard, impermeable materials when friction was presented as a function of sliding speed, lubricant viscosity, and normal load (Fig 1, dashed line)[25]. At low speeds and viscosities, and high loads, contacting asperities support the normal stress, and consequently friction and wear are high–called boundary mode lubrication. As speed and viscosity increase or load decreases, a film of interfacial fluid pressurizes reducing the stress on asperities. In the classical analysis with hard, impermeable materials with specific geometries, the curve mapped out a friction transition as interfacial fluid pressurized until the surfaces were fully separated–called hydrodynamic lubrication. Recently, it was shown that soft, permeable materials undergo similar transitions in lubrication behavior, but do not appear to achieve full hydrodynamic lubrication[26]. It was hypothesized that these transitions resulted from fluid pressurization, and that these deviations from the classical Stribeck behavior were due to fluid flow into and out of the contacting surfaces. This transition away from the classical curve was coined the elastoviscous transition (Fig 1, solid line)[26].

In this study, we apply this framework of lubrication to articular cartilage by mapping its elastoviscous transition. To accomplish this, we performed experiments with Sommerfeld number ranging 7 orders of magnitude by changing both sliding speed and viscosity. This framework enabled calculation of maximum and minimum friction, and a key parameter describing the transition between them. Carrying out these tests in combinations of lubricin and HA enabled assessment of their independent contributions to the elastoviscous transition curve. In addition, our studies revealed a synergistic mechanism in which HA is localized to the articular surface by lubricin that drives the system towards a low friction regime. Importantly, this synergy was not specific to HA, but was replicated by another viscous polymer dextran, albeit at much higher concentrations. As such, our studies suggest a new strategy for designing lubricating systems.

## Results

### Elastoviscous Transitions of Cartilage

**Fig 2:**
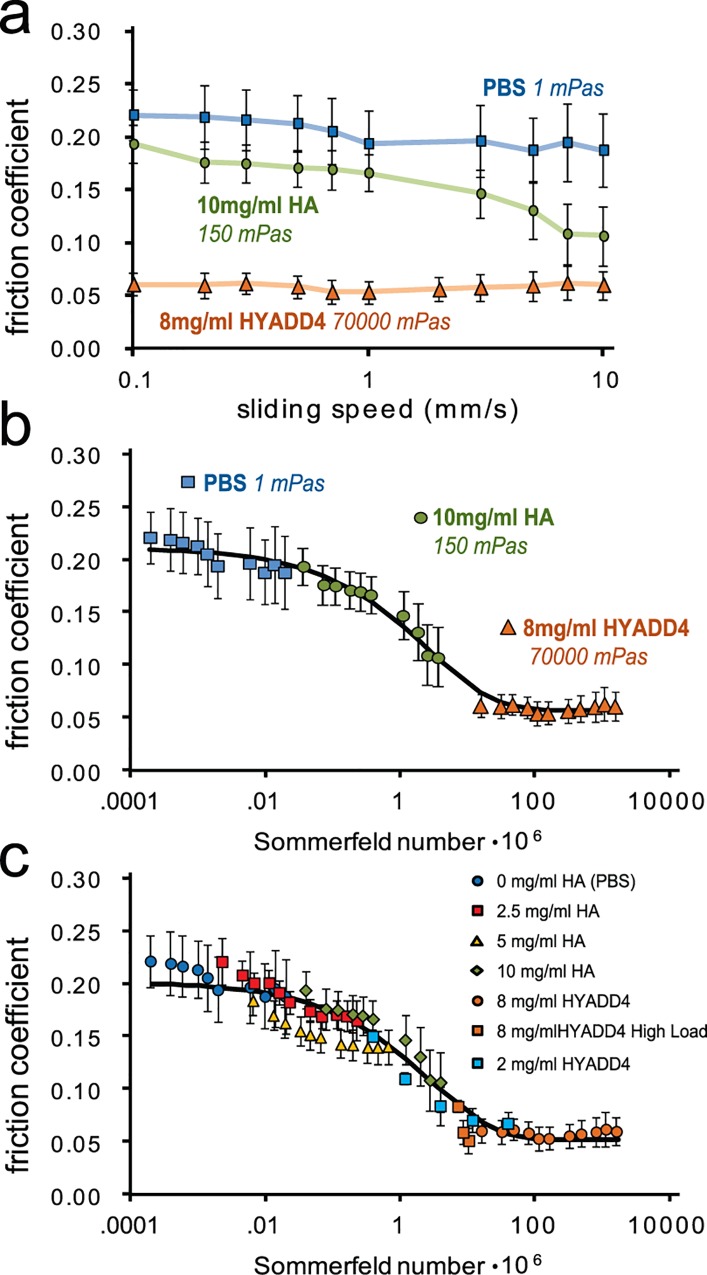
Presentation of friction coefficient as a function of the Sommerfeld number reveals viscous lubrication by HA. (Top) Friction coefficient as a function of sliding speed for three different lubricants with a wide range of viscosities provided a range of friction coefficients that span almost an order of magnitude (n = 5). (Middle) When the friction coefficients from the top panel are presented as a function of the Sommerfeld number (Eq 1) instead of sliding speed, the mechanisms of lubrication become apparent and are fit to a model curve (Eq 2) to determine alterations of the elastoviscous transition. (Bottom) Serial dilutions of the HA solution provided overlapping data sets confirming HA had no influence on the boundary friction coefficient, and increased load (4.2, 5.2, and 6.2 N; all at 0.1 mm/s) and decreased concentration (2 mg/ml) tests with HYADD4 revealed convergence of HA and HYADD4 in the transition region (HYADD4 2 mg/ml and high load, n = 4, all others n = 5) (data points represent mean±SEM).

In order to measure lubrication mode transitions of articular cartilage, friction coefficients of cartilage on glass were collected over speeds ranging 2 orders of magnitude while bathed in lubricants with viscosities varying 5 orders of magnitude (Fig 2A). The lubricants used were phosphate buffered saline (PBS, 1 mPa s dynamic viscosity), 700 kDa HA at 10 mg/mL (150 mPa s), and a hydrophobic HA derivative (HYADD4; 72,000 mPa s)[27]. The lubricants with elevated viscosities provided lower friction coefficients for unaltered cartilage surfaces (i.e., surfaces containing endogenously bound lubricin) (Fig 2A). Notably, HA reduced friction coefficients at elevated sliding speeds. This same data, was then presented as a function of the dimensionless Sommerfeld number[26,28], *S*:

S=VηaFn (1)

where *V* is sliding speed, *η* is lubricant viscosity, *a* is contact width (6 mm), and *F*_*n*_ is normal load (2.6 ± 0.11 N, corresponding to a 92 ± 4 kPa normal stress). Such analysis revealed *μ* to be a continuous function of *S* for all studies, which included changes in lubricant composition and concentration, sliding speed, and normal load (Fig 2B and 2C). Reduced friction as a function of *S* was classically described by the formation of a pressurized fluid film between hard bodies in contact [25], but notably, for soft, permeable contacts, even though asperities may not fully separate, *μ* can still be described as a continuous function of *S* [26]. In such systems, it has been observed that these friction reductions can result from the formation of a viscous film[29], and we have previously reported on similar friction decreases in this system at increased speed or decreased load for cartilage bathed in PBS[30]. Further, we examined dilutions of HA at 5 and 2.5 mg/mL to examine the convergence of the HA solutions towards the boundary mode plateau and found no differences at low *S* between PBS and HA. This finding confirmed previous reports that HA has no effect on the boundary mode friction coefficient of healthy cartilage (Fig 2C)[3,11,22]. We also noted that HA and HYADD4 data converged near the transition to minimum friction and dilution of HYADD4 to 2 mg/mL confirmed viscous lubrication by HYADD4. The data were fit to a curve[31] where friction coefficient, *μ*, transitioned from a boundary friction coefficient, *μ*_*B*_ (the value of *μ* at *S = 0)*_,_ to a minimum friction coefficient, *μ*_*min*_ (the value of *μ* at *S = ∞*), and allowed the determination of a transition number, *S*_*t*_, that is representative of the Sommerfeld number at the midpoint of the transition. This equation was given by:

$$\mu (S)=\mu_{min}+(\mu_{B}-\;\mu_{min})e^{-{\left(S/S_{t}\right)}^{d}}$$ (2)

where *d* is a fitting parameter controlling the slope of the transition zone. The data (both Fig 1B and 1C) fit the curve well with a coefficient of variation of the RMS error below 0.08 and curve fit coefficients that differed by less than 7% between the two data sets. Collectively, these data revealed that cartilage transitioned smoothly from high to low friction modes as a function of the Sommerfeld number, consistent with an elastoviscous transition previously described for soft, permeable materials [26].

### Synergy Between Lubricin and HA

**Fig 3:**
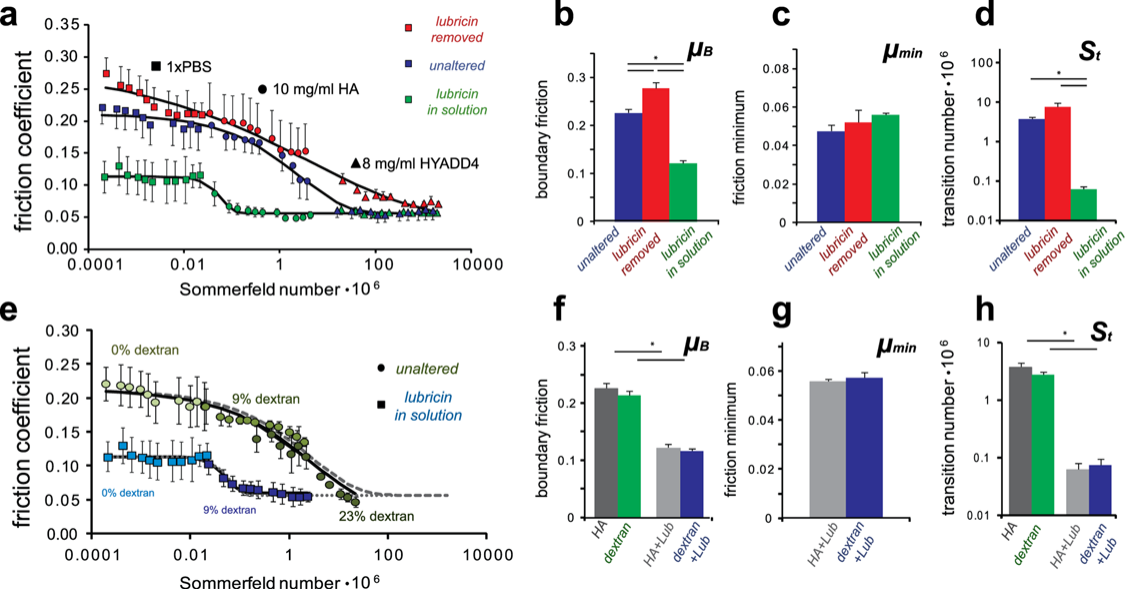
(A) Altering the presence of lubricin at the articular surface and in the lubricant solution revealed distinct elastoviscous transition curves. (B-D) The curve fit parameters revealed the importance of lubricin in boundary lubrication and also its importance in facilitating the transition away from boundary mode lubrication when present with HA. (E-H) Replication of the HA curves by dextran revealed that both the viscous lubrication by HA and the synergy with lubricin are not specific to the chemistry of HA. (n = 5 for unaltered, n = 3 for lubricin removed and lubricin in solution; data points represent mean±SEM).

Friction transitions were mapped for two other lubricin conditions in addition to the unaltered cartilage surfaces (Fig 3A). Samples with lubricin removed from the articular surface[13,32,33] produced a similarly shaped transition curve, but the boundary friction coefficient (*μ*_*B*_) increased 27% from 0.22 to 0.28 (p < 0.05; Fig 3B). The transition number (*S*_*t*_) increased from 3.7 10^−6^ to 7.6 10^−6^ (p = 0.10; Fig 3D) and there was no noticeable increase in the minimum friction coefficient (*μ*_*min*_) (Fig 3C), indicating that minimum friction may be dependent on other factors such as counterface roughness or permeability. Interestingly, in the absence of lubricin, cartilage lubricated by HYADD4 achieved *μ*_*min*_ only at elevated speeds.

Addition of 20 μg/mL full-length recombinant lubricin[13,32] lowered the boundary friction coefficient more than a factor of 2 (μ_B_ = 0.12, p < 0.05) (Fig 3B), a result previously reported for this concentration of lubricin[32]. More interestingly, the transition number decreased 2 orders of magnitude (*S*_*t*_ = 0.06 10^−6^) (p < 0.05; Fig 3D). Consequently, HA solutions also yielded *μ*_*min*_ at relatively low sliding speeds (*V* > 0.3 mm/s). While the mechanism of viscous boundary lubrication has been used to explain similar observations in salivary mucins[29], our studies indicate this theory of lubrication is also applicable to articular cartilage. In essence, lubricin may act to localize HA near the articular surface, locally increase the viscosity, and drive the surfaces away from boundary mode lubrication. This idea of increasing local viscosity would not be expected from bulk rheology measurements where lubricin decreases viscosity by a factor of ~3 when added to HA solutions[34], but may be indicative of a mechanism of confined fluid which has been shown to increase measured viscosities of synovial fluid by orders of magnitude[35].

### Lubricin-HA Synergy is not Specific to HA Chemistry

Lubricin and HA together facilitated the transition away from boundary mode lubrication, but the question remains whether this interaction is specific to these molecules. To answer this question, the above experiments were repeated, replacing HA with another viscous polymer, 2 MDa dextran (Fig 3E–3H). For the unaltered cartilage surfaces, the friction transition curves were similar for both dextran and HA. Notably, even 23% dextran (w/v) was not able to induce minimum friction even at the highest testing speeds. In the presence of exogenous lubricin, dextran again produced similar lubrication curves to HA. Collectively, these data show that HA and dextran produce similar elastoviscous transitions with μ_B,_ μ_min,_ and *S*_*t*_ coefficients that differ by less than 25%. As such, these data indicate that the viscous lubrication by HA and interaction with lubricin at the cartilage surface is not specific to the chemistry of HA. Due to the similarities between the HA and dextran curves, it is likely this phenomenon is mechanically motivated and could arise from entanglement, or hydrophobicity/hydrophilicity. While it is possible that similarities between HA and dextran as viscous polysaccharides are important to this interaction, it is noteworthy that this interaction with lubricin can occur with other molecules than HA.

### Lubricin Facilitates the Aggregation of HA at the Articular Surface

**Fig 4:**
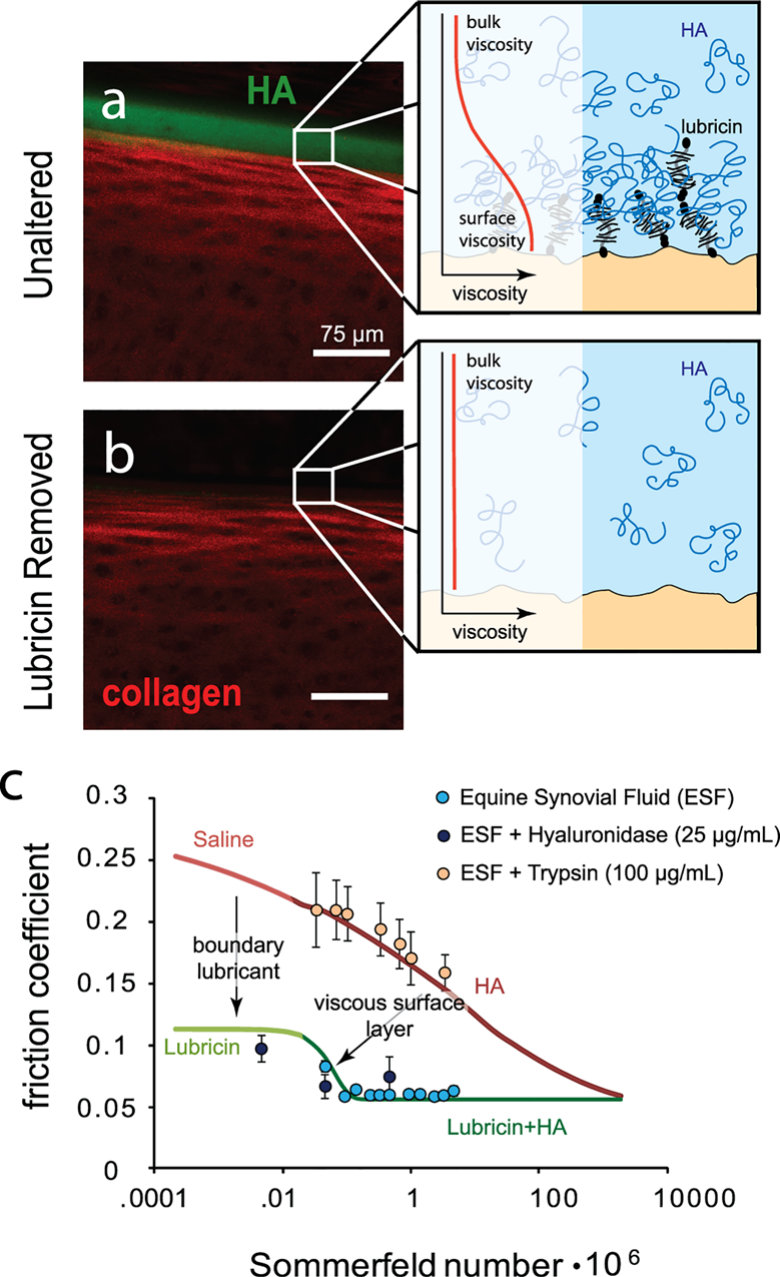
In native cartilage, lubricin bound to the surface facilitates HA aggregation near the surface (A). Lubricin likely entraps HA through entanglements, causing a local increase in viscosity near the tissue surface (A, inset). When lubricin is removed from the surface, HA does not aggregate (B) and surface viscosity is likely similar to the bulk (B, inset). Boundary lubrication by lubricin shifts the boundary regime down and increased viscosity near the surface shifts the elastoviscous transition such that the low friction regime occurs at lower sliding speeds consistent with viscous boundary lubrication (C). These phenomena of lubricin and HA replicate the lubrication by synovial fluid which transition to low friction at low speeds. By treating synovial fluid with hyaluronidase, friction is shifted back towards the boundary mode plateau as the fluid viscosity decreases, and trypsin treatment disrupts the synergy between lubricin and HA by digesting lubricin (n = 4; data points represent mean±SEM).

To further test the hypothesis that lubricin enhances lubrication through localizing HA near the surface, we imaged articular cartilage with and without lubricin after exposure to HA. Specifically, unaltered cartilage samples were incubated in a fluorescein-tagged HA solution, tapped dry and viewed on a confocal microscope. Simultaneous imaging of both the extracellular matrix through confocal reflectance and the labeled HA, revealed that HA aggregated at the articular surface (Fig 4A), but this aggregation was absent when lubricin was removed from the surface (Fig 4B). We also found that the aggregation of HA at the surface was weak, as the presence of HA at the lubricin-containing surfaces disappeared after a short rinse with physiologic saline. The weak nature of this interaction can explain why HA appears to have no effect on the boundary friction coefficient and why its results are replicable by another biopolymer.

### Lubricin-HA Synergy Replicates Lubrication by Synovial Fluid

There are a multitude of other proposed molecular lubricants in synovial fluid (e.g., phospholipids, gamma globulin, etc.)[5,36]. Consequently, to determine whether this interaction between lubricin and HA replicates the full lubricating effect of synovial fluid, subsequent tests were performed using intact equine synovial fluid, as well as synovial fluid depleted of HA by hyaluronidase[22] and depleted of lubricin by trypsin[34] (Fig 4C). For synovial fluid incubated with hyaluronidase (20 mPa s viscosity), friction coefficients approached the boundary mode plateau at slow sliding speed and transitioned toward the minimum friction at elevated speed, with little indication that the results diverged from the previously found curve for lubricin in solution (Fig 4C). In contrast, trypsin treatment had little effect on viscosity (146 mPa s) but yielded data consistent with curves for cartilage in the absence of lubricin. Thus, in agreement with the above studies using recombinant molecules (Fig 3), we find that in synovial fluid HA provides elevated viscosity to transition away from boundary mode, lubricin alters boundary mode friction, and the synergistic mechanism between them reduces the viscosity necessary to transition away from boundary mode.

## Discussion

### Lubricin-Mediated Synergy

In this paper, we identified an interaction between lubricin and HA that synergistically enhances lubrication of articular cartilage. This synergy between HA and lubricin is dependent on the binding of lubricin to surfaces and is not specific to the chemistry of HA. Other theories of lubricin-HA synergy at the articular surface exist[18,19,24,37], but we have shown here that together, lubricin and HA are effective by forcing the transition away from the relatively higher friction associated with the boundary mode of lubrication. Recently, Das *et al* showed that adsorbed HA on mica surfaces interacted with lubricin and prevented wear, likely by forming a viscous gel layer at the surface[19]. This gel layer was estimated to be four orders of magnitude more viscous than the free solution. Similarly, Greene *et al* theorized that the chondroprotective basis of synovial fluid lubrication was mechanically driven[18] by trapping of HA near the surface and subsequent aggregation and cross-linking with lubricin. In this study we build upon these mechanically motivated gel-layer theories, and provide evidence that lubricin binding to articular surfaces initiates this mechanism with HA.

This theory of surface protection and lubrication by formation of a gel layer is not specific to HA and lubricin, and it has been previously referred to as “viscous boundary lubrication”[29]. This mechanism of lubrication was observed for the aggregation of salivary mucins. With viscous, protein-adsorbed layers at sliding interfaces, the transition away from boundary mode lubrication may not scale with bulk solution rheology, but instead scale with the viscosity of the adsorbed layers [29]. In a similar manner to salivary mucins, we note that a surface gel layer of lubricin and HA could be a factor of 100 times more viscous than the bulk solution (Fig 4A inset; based on the alterations of *S*_*t*_) which serves to reduce the operating time of a joint in boundary mode lubrication.

Lubrication by a gel layer in synovial fluid is distinct from the mechanism for salivary mucins, which appears to be mediated by protein aggregation. The present study indicates that sufficient lubrication of mammalian joints is dependent on the interaction of two molecules with different structures. Lubricin can be thought of simply as an amphiphillic tethered polymer brush that interacts in a non-specific manner with HA, which can be thought of as a linear viscous polymer. Whether this interaction is purely a mechanical entanglement, or it is dictated by the hydrophobic and hydrophilic nature of these molecules remains a subject of further exploration. The results presented here indicate that this interaction facilitates the formation of a gel-like layer at the articular surface where optimal lubrication is achieved by the cooperation of two molecules, lubricin and HA.

### The Role of HA Viscosity in Cartilage Lubrication

In a classical analysis of rheology, HA solutions have exhibited significant shear thinning when tested using model shear configurations (e.g., metal plates or couettes)[38,39]. This rheological behavior led previous researchers to note that friction reduction through increased normal stresses would be overshadowed by shear thinning. Similarly, shear thinning behavior has been observed for synovial fluid as well[34,40]. Recently; however, it was shown that microstructurally, synovial fluid may behave very differently when confined[35]. Banquy *et al*. showed orders of magnitude increases in viscosity of synovial fluid when confined between mica surfaces. Similarly in this study, the friction trends appeared to scale with the viscosities at low shear rates, which would not be expected if significant shear thinning occurred at the tissue surface. However, the affinity of HA to the articular surface may prove to be a crucial mechanism in restricting shear thinning or possibly in facilitating increased viscosity in a surface gel layer, as the molecules may behave similarly to the confined synovial fluid[35].

### Divergence from the Classical Stribeck Curve

The Stribeck curve has been used for more than a century to describe lubrication modes of both engineering machines and the natural mechanisms at play in mammalian joints. However, recent experiments and theories of hydrogel lubrication proposed a divergence from the classical curve, which assumes full separation of solid asperities at high values of a lubricant parameter (e.g. the Sommerfeld number)[26]. Due to the compliance of soft materials like cartilage, it is possible that localized pools of pressurized fluid may form and reduce friction but not fully separate the surfaces in cases where pressurized fluid may flow into and out of the contact, a phenomenon that may be dictated by pore size or permeability[26]. In this study, we observed a transition away from the relatively higher boundary mode lubrication consistent with mixed-mode lubrication, but friction coefficients reached minimum values (μ > 0.04) above what would be expected for true hydrodynamic friction (μ << 0.01). Nevertheless, these data demonstrate cartilage lubrication over 7 orders of magnitude in *S* that is consistent with an elastoviscous transition. Further studies may reveal what role the mechanics of the articular cartilage play in the transitions outlined above and what implications degradation of the extracellular matrix may have on lubrication mechanisms.

### Limitations

While this study establishes the framework for studying the elastoviscous transition in cartilage lubrication, there are several limitations to be discussed. Although the concentration of lubricin used in this study was sub-physiologic, the level of lubrication observed, although not identical, was largely comparable to that of saturating levels of lubricin (μ = 0.12 ± 0.01 at 20 mcg/ml in the current study vs μ = 0.10 ± 0.01 at 300 mcg/ml reported previously [32]). Nevertheless, further studies should be aimed at elucidating the role of lubricin concentration on the lubrication mechanisms found in this study.” Further, this study utilized a stationary contact area configuration[41] where polished glass was used as the counterface. Notably, boundary friction coefficients for PBS, lubricin, and synovial fluid are similar in this system to a cartilage-cartilage bearing[42], but further studies may be aimed at analyzing the synergistic mechanism found in this study for configurations of cartilage on cartilage or cartilage on meniscus. Lastly, the Sommerfeld number is a function of normal load in addition to sliding speed and viscosity (Eq 1), and while increases in friction coefficient were observed at higher loads (Fig 2C), load was not studied extensively for all lubricant formulations. Consequently, a systematic evaluation of the contributions of load to the elastoviscous transition can solidify this system as a robust tool to study the lubrication mechanisms of cartilage and other soft material bearings.

### Implications for Joint Injury, Disease, and Therapy

This study shows that articular cartilage lubrication can be described as a smooth continuous function of the Sommerfeld number, and cartilage likely experiences lubrication throughout a wide range of Sommerfeld numbers. High *S* can occur during the unloaded swing phase of gait (high relative velocity and low load), and low *S* may can occur during foot strike (low relative velocity and high load). Additionally, changes in viscosity affect *S*, and this is particularly important in joint injury and disease, where the composition of synovial fluid changes significantly[10,12,43]. Notably, HA concentration and size, as well as lubricin concentration, are altered for extensive periods of time after the initiation of trauma or disease. As such, both of these molecules have been targets for joint therapy, with HA injections being used clinically for decades[20] and multiple recent studies demonstrating disease-modifying capabilities of lubricin injections in animal models of joint injury[14,44]. The experimental and analytical framework used in this study gives unique insights into the mechanisms of action of these therapies. First, mapping the elastoviscous transition makes it clear that HA is an exceptional viscous lubricant, with modified forms (HYADD4) producing superior lubrication to dextrans that are more than double in size and are present in over 30 fold higher concentrations. Secondly, it is clear that localization of HA by lubricin synergistically enhances lubrication, suggesting that lubricin delivery to joints may be an important therapy, both alone or in combination with HA. Collectively, these data point to the importance of studying the regulation of both of these molecules in acute and chronic diseases to more fully understand the pathogenesis of arthritis.

More broadly, the identification of this lubrication mechanism that relies on the cooperation of two different lubricating molecules to protect soft surfaces from high friction and wear is applicable outside of the realm of biology. Through creation of biomimetic systems that exploit this naturally occurring mechanism, scientists can create more efficient bearing systems of soft material interfaces.

## Methods

### Tribological Testing

Friction coefficients were measured on our previously described, custom-built tribometer [30,32,33]. Cartilage samples were obtained from the patellofemoral groove of neonatal (1–3 day old) bovine stifles. Cylindrical cartilage samples (6 mm in diameter by 2 mm high) were mated against a polished glass flat counterface while bathed in a lubricant bath in a configuration consistent with a tilt pad bearing (See: Gleghorn *et al*. 2008). Samples were tested in a stationary contact area configuration that mitigates the effects of interstitial fluid pressurization on friction coefficient measurements [33,41]. Before friction testing, samples were compressed to 25% strain and allowed to depressurize over the course of 1 hour resulting in average normal loads of 2.6 N. After fluid pressure dropped to the ambient pressure, the glass counterface was reciprocated at predetermined speeds ranging from 0.1 to 10 mm/s. Friction coefficients were recorded as the ratio of shear load to normal load measured by a biaxial load cell. Coefficients were calculated at the end of sliding when friction had reached an equilibrium value and averaged for both the forward and reverse sliding directions[45].

### Lubricant Formulations and Cartilage Surfaces

The role of hyaluronic acid and viscosity were analyzed using three different HA conditions within the lubricant bath. A HA-free control bath was phosphate buffered saline (PBS; Corning, Manassas VA). Sodium hyaluronate (Fidia Farmaceutici, Padua Italy) with 500–730 kDa molecular weight obtained from *Streptococcus Equi* fermentation and formulated to a final solution of 10 mg/mL in PBS was used as the HA solution. HYADD4 (Fidia Farmaceutici, Padua Italy) which is a partially hydrophobic hexadecyl derivative of HA with a 3% mol/mol repeating unit substitution provided a lubricant bath with increased viscosity at a concentration of 8 mg/mL in PBS[27,46]. These three lubricant baths were also tested with and without rhLubricin added into solution at a concentration of 20 μg/mL (a gift from Dr. Carl Flannery, Pfizer). The 20 μg/mL concentration was chosen as we have previously shown a saturation of boundary lubrication in this system for this concentration[32]. For the baths without lubricin, two cartilage surface conditions were tested. First cartilage samples were tested unaltered after dissection containing their endogenously bound lubricin, and samples were also tested after lubricin was removed from the articular surface via a hypertonic (1.5M) saline incubation for 25 minutes followed by a 1 hr re-equilibration in PBS[13]. The efficacy of lubricin extraction was analyzed via immunohistochemical staining (S1 File and S1 Fig). For the lubricant baths containing lubricin, only unaltered cartilage surfaces were tested.

Subsequent tests using 2 MDa dextran (Sigma Aldrich, St Louis MO) in place of HA were carried out using cartilage with unaltered surfaces using 9% (w/v) and 23% solutions and also 9% solution with 20 μg/mL rhLubricin added. Finally, tests using equine synovial fluid from the carpus joints of skeletally mature horses provided a comparison of the solutions to the native synovial fluid. To determine the role of synovial fluid viscosity, equine synovial fluid (ESF) was also tested after two hour incubation with 25 μg/mL bovine testes hyaluronidase (Sigma Aldrich, St Louis MO) added into the solution[22]. To determine the role of lubricin, ESF was also digested with TPCK trypsin (Sigma Aldrich, St Louis MO) from bovine pancreas as previously reported[34]. Briefly, 100 μL of 2 mg/mL trypsin was added to ESF for 2 hours at 37 C under constant stirring conditions.

### Rheological Testing

To determine the role of viscosity, a commercial rheometer (TA Instruments DHR3 Rheometer, New Castle DE) was used to measure the low shear rate viscosity of the lubricant baths. For most lubricants, a 40 mm diameter cone-plate set up with a 2° angle was used at a shear rate of $\dot{\gamma }={1\;s}^{-1}$. For HYADD4 a custom sandblasted 25mm plate-plate set up was used to mitigate wall slip due to elevated viscosity. Viscosities were measured of 156 mPas for 10 mg/mL HA, 72,000 mPas for 8 mg/mL HYADD4, 200 mPas for equine synovial fluid, 20 mPas for ESF incubated with hyaluronidase, 146 mPas for ESF incubated with trypsin, 108 mPas for 9% dextran, and 884 mPas for 23% dextran. Subsequent serial dilutions of the HA solutions provided viscosities of 30 mPas and 11 mPas for 5 mg/mL and 2.5 mg/mL solutions, respectively, and dilution of HYADD4 to 2 mg/mL provided a viscosity of 1400 mPas.

### Confocal Imaging

Articular cartilage explants both containing the endogenously bound lubricin and with endogenous lubricin removed were incubated in 1 mg/mL 750 kDa fluorescein-tagged HA (Creative PEG Works, Winston Salem NC) that was dialyzed overnight to remove any unbound label for 5 minutes. After incubation, samples were cut into hemicylinders, tapped dry on a glass slide and a cross-sectional view was imaged on a Zeiss LSM710 confocal microscope. Both the fluorecein signal and reflectance from the collagen extracellular matrix were merged in images to view whether affinity of HA to the articular surface is dictated by localized lubricin.

### Statistical Analysis

To determine the uncertainty in the curve fit parameters of importance (i.e., boundary friction, minimum friction, and the Sommerfeld number at the mid-point along the transition), a Monte Carlo simulation was conducted on a point-by-point basis based on the standard deviation of measurements and a random normal distribution with a mean of 0 and a standard deviation of 1 [45,47]. To determine differences in curve fit parameters between groups, a one way ANOVA was conducted and significance was set at p < 0.05. Data and error bars represent mean ± SEM.

## Supporting Information

S1 Fig The hypertonic saline incubation for 25 minutes effectively removes lubricin from this tissue source. The cartilage surfaces were not structurally altered as revealed by proteoglycan staining (Saf-O), collagen staining (Picrosirius Red), and collagen organization (Picrosirius Red viewed under polarized light).
(TIF)

S1 File Verification of lubricin removal using hypertonic saline. (DOC)
