# Supplementary figures and images for: Elastoviscous Transitions of Articular Cartilage Reveal a Mechanism of Synergy between Lubricin and Hyaluronic Acid
Source: PLoS One. 2015 Nov 24;10(11):e0143415. doi: 10.1371/journal.pone.0143415 (PMC4658013; doi:10.1371/journal.pone.0143415)

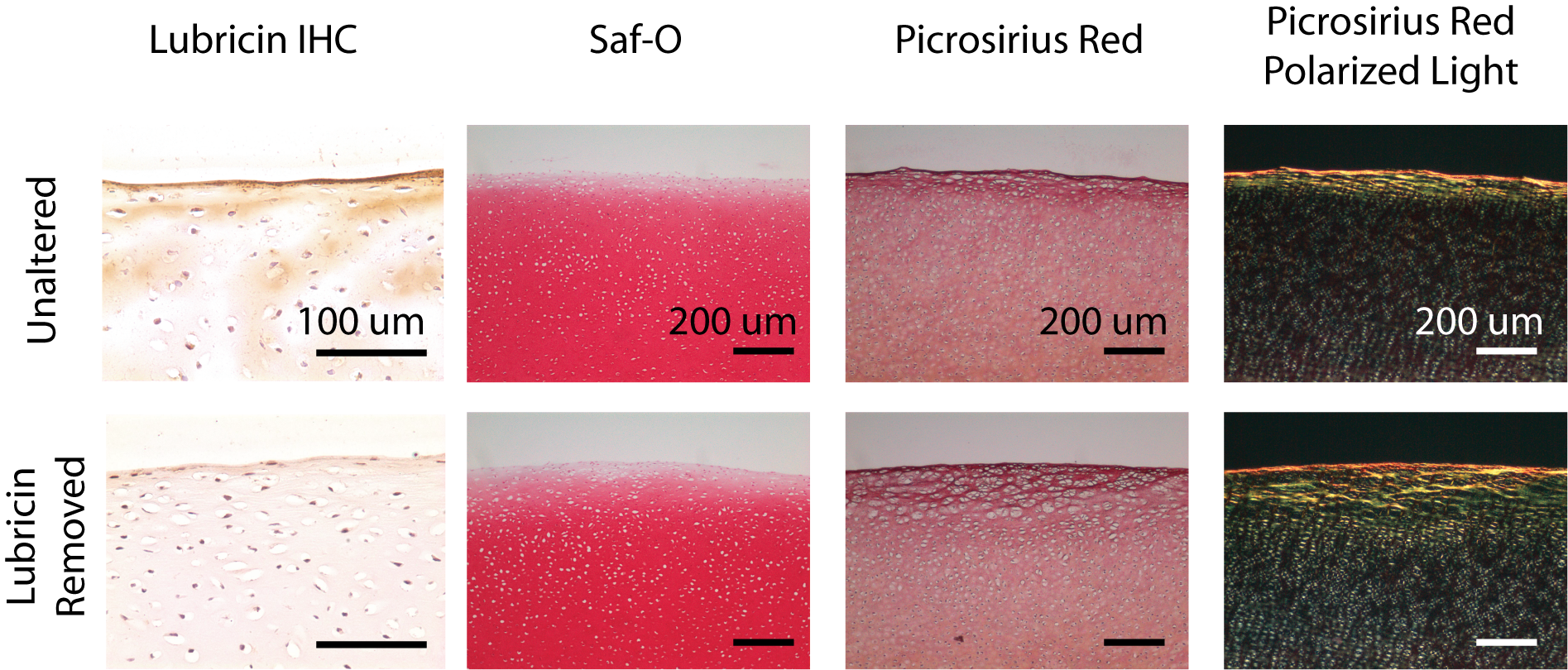

Supplement: S1 Fig — The cartilage surfaces were not structurally altered as revealed by proteoglycan staining (Saf-O), collagen staining (Picrosirius Red), and collagen organization (Picrosirius Red viewed under polarized light). (TIF) [file pone.0143415.s001.tif]
