# Supplementary material for: Elastoviscous Transitions of Articular Cartilage Reveal a Mechanism of Synergy between Lubricin and Hyaluronic Acid
Source: PLoS One. 2015 Nov 24;10(11):e0143415. doi: 10.1371/journal.pone.0143415 (PMC4658013; doi:10.1371/journal.pone.0143415)
Supplement: S1 File — (DOC) [file pone.0143415.s002.doc]

**Supplemental Material**

The efficacy of the 1.5M hypertonic saline incubation was analyzed via immunohistochemical staining using a commercially available lubricin antibody (Abcam; ab28484). To confirm previous studies that the rest of the tissue is unperturbed, Safranin O staining and Picrosirius red staining was also conducted to show no effects on both proteoglycan and collagen content and localization at the articular surface, respectively.

Briefly, cartilage was left unaltered or incubated in 1.5 M NaCl for 25 minutes followed by a 60 minute equilibration in PSB. Samples were then fixed in buffered formalin, embedded in paraffin blocks, and sectioned. For immunohistochemistry, a previously reported procedure was followed^33^. Briefly, antigen retrieval was conducted with citric acid (pH 6) at 90 C for 20 minutes, followed by two washes in TRIS buffered saline with 0.5% TWEEN-20 (pH 7.4), 30 minute incubation with 3% hydrogen peroxide, 60 minute incubation with a blocking serum containing normal serum, bovine serum albumin, TWEEN-20, and Triton X100. Between each step slides were washed in PBS twice for 5 minutes each. An overnight incubation with the primary antibody was conducted at 4°C in humidity chambers. The secondary antibody (Vector, Burlingame CA) was applied for 30 minutes and a 30 minute incubation in an avidin-biotin complex (Vector) followed. Samples were stained using a peroxidase substrate (ImmPACT DAB) and counterstained with hematoxylin (Vector). Samples were also stained with Safranin O and Picrosirius red.

The histologic analysis (SFig 1) revealed that hypertonic saline incubation was effective at removing surface lubricin for this neonatal tissue source while leaving both the proteoglycan and collagen structures of the tissue relatively unperturbed.

**Supplemental Figure Caption**

Supplemental Figure 1: The hypertonic saline incubation for 25 minutes effectively removes lubricin from this tissue source. The cartilage surfaces were not structurally altered as revealed by proteoglycan staining (Saf-O), collagen staining (Picrosirius Red), and collagen organization (Picrosirius Red viewed under polarized light).
